# Population pharmacokinetics and exposure–response modeling and simulation for evolocumab in healthy volunteers and patients with hypercholesterolemia

**DOI:** 10.1007/s10928-018-9592-y

**Published:** 2018-05-07

**Authors:** Mita Kuchimanchi, Anita Grover, Maurice G. Emery, Ransi Somaratne, Scott M. Wasserman, John P. Gibbs, Sameer Doshi

**Affiliations:** Amgen Inc., One Amgen Center Dr, Thousand Oaks, CA USA

**Keywords:** Evolocumab, PCSK9, Population pharmacokinetics, Exposure–response, Monoclonal antibody, Hypercholesterolemia

## Abstract

Evolocumab, a novel human monoclonal antibody, inhibits proprotein convertase subtilisin/kexin type 9, a protein that targets low-density lipoprotein-cholesterol (LDL-C) receptors for the treatment of hyperlipidemia. The primary objective of this analysis was to characterize the population pharmacokinetics (popPK) and exposure–response relationship of evolocumab to assess if dose adjustment is needed across differing patient populations. Data were pooled for 5474 patients in 11 clinical studies who received evolocumab doses of 7–420 mg at various frequencies, either intravenously or subcutaneously. Evolocumab area under concentration–time curve from 8 to 12 weeks (AUC_wk8–12_) was simulated for individuals using the popPK model and was used to predict the LDL-C response in relation to AUC_wk8–12_. Evolocumab was eliminated through nonspecific (linear) and target-mediated (nonlinear) clearance. PopPK parameters and associated variabilities of evolocumab were similar to those of other monoclonal antibodies. The exposure–response model predicted a maximal 66% reduction in LDL-C from baseline to the mean of weeks 10 and 12 for doses of evolocumab 140 mg subcutaneously every 2 weeks or 420 mg subcutaneously once monthly. After inclusion of statistically significant covariates in an uncertainty-based simulation, LDL-C reduction from baseline at the mean of weeks 10 and 12 was predicted to be within 74% to 126% of the reference patient for all simulated patient groups. Evolocumab had nonlinear pharmacokinetics. The range of responses based on intrinsic and extrinsic factors was not predicted to be sufficiently different from the reference patient to warrant evolocumab dose adjustment.

## Introduction

Reduction of low-density lipoprotein-cholesterol (LDL-C) is a primary target for pharmacotherapy in patients with cardiovascular disease [1]. LDL receptor (LDLR) recycling plays a critical role in the maintenance of cellular and whole-body cholesterol balance by regulating plasma LDL-C levels [2]. Proprotein convertase subtilisin/kexin type 9 (PCSK9) binds to the LDLR and promotes degradation of the LDLR within hepatocytes, thereby decreasing the recycling of LDLR and increasing LDL-C concentrations [3, 4]. Several studies report that statins increase PCSK9 [5–7], which may attenuate the cholesterol-lowering effect of statins [8]. Therefore, use of a PCSK9-antagonizing therapy is a particularly effective strategy in combination with a statin to lower LDL-C and improve dyslipidemia.

Evolocumab is a fully human monoclonal immunoglobulin G2 antibody that binds to human PCSK9 with high specificity and prevents the interaction of PCSK9 with LDLR. Inhibition of PCSK9 by evolocumab leads to increased LDLR expression and decreased circulating concentrations of LDL-C [9]. In phase 1, 2, and 3 clinical studies, evolocumab was administered subcutaneously (SC) at doses ranging from 7 to 420 mg, either alone or in combination with statins. At doses of at least 140 mg, there is effective PCSK9 inhibition, which translates into a reduction of LDL-C by 54–80% [10].

Following single-dose administration of evolocumab over a wide dose range (7–420 mg) by intravenous (IV) or SC routes in healthy subjects, evolocumab exhibited nonlinear pharmacokinetics (PK) that could be described by parallel linear and nonlinear clearance [11, 12]. After multiple doses of 140 mg SC or higher, the nonlinear target-mediated elimination pathway was nearly saturated, and the linear elimination pathway was dominant [11]. In addition, there was no evidence of time-dependent PK after repeated dosing with the evolocumab regimens that were examined [13]. Randomized controlled studies have shown the efficacy and safety of evolocumab in a variety of patient populations, either in combination with statins or as monotherapy, with significantly greater lipid lowering compared with placebo or ezetimibe [13–23].

Given the diversity of the patient population with hypercholesterolemia, the primary objectives of this analysis were to evaluate the influence of intrinsic and extrinsic covariates as potential sources of variability in the PK and exposure–response relationships of unbound evolocumab using a population approach, and to quantify the effect of these sources of variability on the pharmacodynamic (PD) response to evolocumab.

## Methods

### Clinical studies

This population PK (popPK) analysis of evolocumab pooled data from 11 clinical studies, the relevant characteristics of which are summarized in Table 1. Additional details of the clinical trials are reported elsewhere [11, 13–23].

**Table 1:** Summary of the studies and data included in the analyses

Phase/study	Evolocumab dosing	Study population	Pharmacokinetic sampling	Total patients in study	Total patients in popPK analysis
Phase 1a 20080397	Single dose: intravenous: 21, 420 mg subcutaneous: 7, 21, 70, 210, 420 mg	Healthy subjects	Intensive	56	42
Phase 1b 20080398	Multiple dose: weekly × 6 subcutaneous: 14, 35 mg once every 2 weeks × 3 subcutaneous: 140, 280 mg monthly × 3 subcutaneous: 420 mg	Hypercholesterolemia patients treated with a statin	Intensive	57	43
Phase 2 20090158	Monthly × 3 subcutaneous: 350, 420 mg	Combination therapy in heterozygous familial hypercholesterolemia patients	Trough and PK substudy	167	108
Phase 2 20090159	Monthly × 3 subcutaneous: 280, 350, 420 mg	Combination therapy in statin-intolerant patients	Trough and PK substudy	157	120
Phase 2 20101154	Once every 2 weeks × 6 subcutaneous: 70, 105, 140 mg monthly × 3 subcutaneous: 280, 350, 420 mg	Monotherapy in hypercholesterolemia patients	Trough and PK substudy	361	269
Phase 2 20101155	Once every 2 weeks × 6 subcutaneous: 70, 105, 140 mg monthly × 3 subcutaneous: 280, 350, 420 mg	Combination therapy in hypercholesterolemia patients	Trough and PK substudy	629	463
Phase 3 20110109	Monthly × 12 subcutaneous: 420 mg	Effect durability in hypercholesterolemia patients: monotherapy or combination therapy	Sparse	901	598
Phase 3 20110114	Once every 2 weeks × 6 subcutaneous: 140 mg monthly × 3 subcutaneous: 420 mg	Monotherapy in hypercholesterolemia patients	Sparse	614	293
Phase 3 20110115	Once every 2 weeks × 6 subcutaneous: 140 mg monthly × 3 subcutaneous: 420 mg	Combination therapy in hypercholesterolemia patients	Sparse	1896	1081
Phase 3 20110116	Once every 2 weeks × 6 subcutaneous: 140 mg monthly × 3 subcutaneous: 420 mg	Combination therapy in statin–intolerant patients	Sparse	307	187
Phase 3 20110117	Once every 2 weeks × 6 subcutaneous: 140 mg monthly × 3 subcutaneous: 420 mg	Combination therapy in heterozygous familial hypercholesterolemia patients	Sparse	329	210

Analyses were based on pooled data from 5474 patients, including 3414 who received evolocumab and were included in the final popPK analysis and 1312 from 4 phase 2 studies who were included in the exposure–response analysis

### Bioanalytical assay

For all clinical studies used in this analysis, unbound evolocumab concentrations in human serum were determined using a validated enzyme-linked immunosorbent assay. Microplate wells coated with a mouse anti-evolocumab monoclonal antibody (clone no. 1.18.1, Amgen Inc.) were used to capture evolocumab from serum. The capture reagent incubation wells were then washed, and standards, quality controls, and samples were pipetted into the wells. Unbound materials were removed by a subsequent wash step. Horseradish peroxidase–labeled mouse anti-evolocumab monoclonal antibody (clone no. 1.46, Amgen Inc.) was added to the wells for detection of the captured evolocumab. After another wash step, a tetramethylbenzidine substrate solution reacted with the peroxide and produced a colorimetric signal that was proportional to the amount of evolocumab bound by the capture reagent. The color development was stopped by addition of H_2_SO_4_, and the optical density signal was measured at 450 nm with reference to 650 nm. The lower limit of quantification (LLOQ) and upper limit of quantification for the assay was 0.8 and 10 µg/mL, respectively.

### Software

PopPK and PK/PD data were analyzed using the nonlinear, mixed-effects modeling software program NONMEM (version 7.2) [24] on the NONMEM High Performance Cluster (NONMEM HPC), which is a suite of scripts, procedures, and services that supports popPK and PK/PD analyses. It consists of NONMEM 7.2, NMQual 8.2.7, Subversion 1.6.11, MPICH 3.0.4, Grid Engine 2011.11p1, Intel FORTRAN 13.0.1, R 3.0.1, RStudio 0.97.551, and Perl 5.18.1 (1800). NONMEM jobs in the NONMEM HPC system are run on a Grid Engine moderated pseudo-cluster of Intel^®^ Xeon^®^ CPU X5660 @ 2.80 GHz processors under Red Hat Enterprise Linux 5.8 (Tikanga). Graphical and all other statistical analyses were performed using either TIBCO Spotfire S+ for Windows version 8.2 (TIBCO Software Inc., Palo Alto, CA) or R software 2.10.1 or higher (The R Foundation for Statistical Computing).

### PopPK analysis

**Fig. 1:**
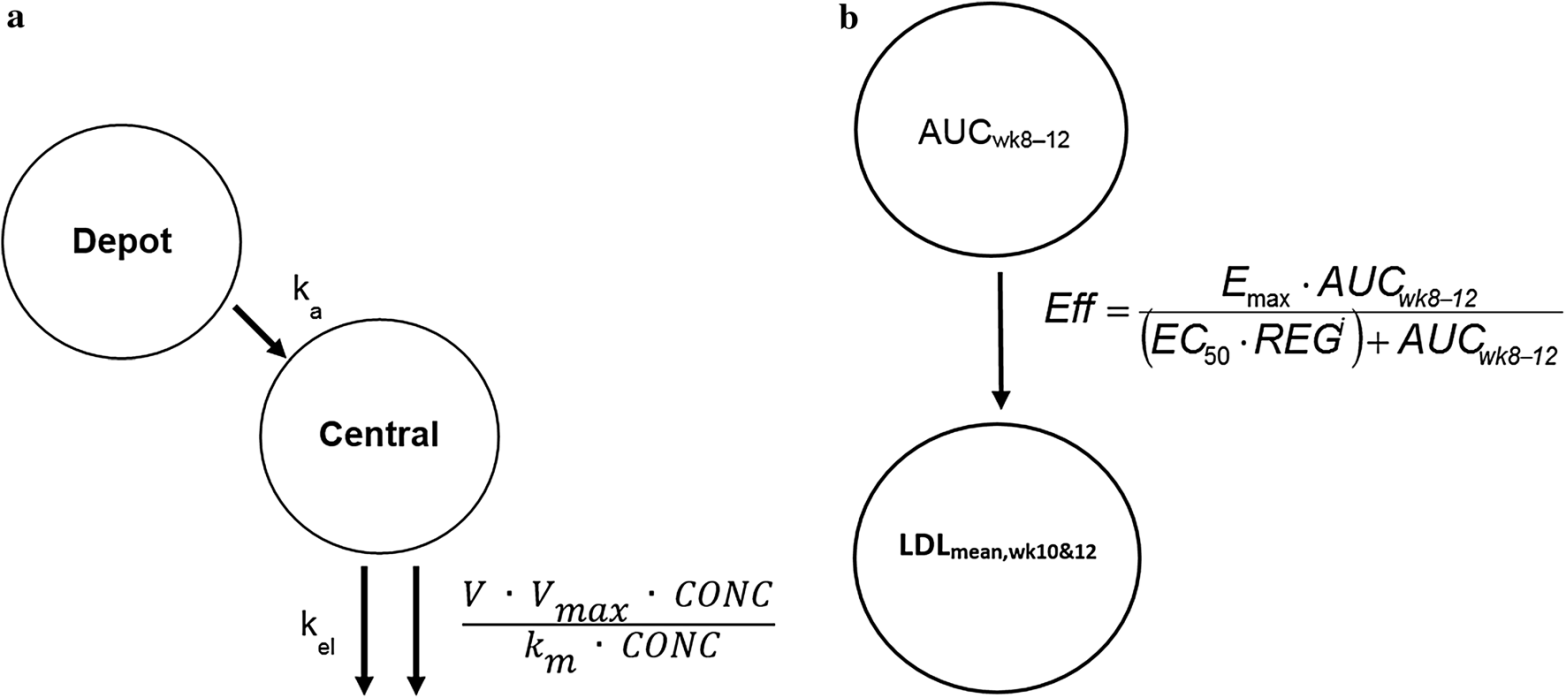
Evolocumab pharmacokinetic and exposure–response model. **a** Pharmacokinetic model; *k*_*a*_ absorption rate constant; *k*_*el*_ elimination rate constant; *k*_*m*_ concentration of half-maximal nonlinear clearance; *V*_*max*_ nonlinear clearance capacity. **b** Exposure–response model; *Eff* LDL-C lowering effect; *E*_*max*_ theoretical maximum evolocumab response for the average of weeks 10 and 12; *EC*_*50*_ ((µg/mL) * day) AUC_wk8–12_ (Q2W) to achieve half-maximal response; *REG* regimen effect on EC_50_ with an indicator variable, *i*, with values of 0 or 1 was used indicate Q2W or QM regimens

Phase 1 and 2 data were used to develop the initial popPK model. A 1–compartment open model with linear and nonlinear elimination pathways from the central compartment (Fig. 1) was parameterized by volume of distribution (V) in the central compartment, linear clearance (CL), and nonlinear clearance (V_max_, k_m_), and was selected based on preliminary analyses and visualizations of the data [25]. Attempts were made to fit a target-mediated drug disposition model [12] using the quasi steady-state (QSS) approximation of the full target-mediated drug disposition (TMDD) model estimating steady state constant (kss) from the data, but results showed that the model was overparameterized given the sparseness of PK data available across subjects (Table 1).

Absorption after SC administration was described by a first-order process from the depot compartment to the central compartment. Bioavailability (F) was used to scale IV to SC dosing. Estimates of the absorption rate constant (k_a_) and F were fixed from the phase 1a, densely sampled, single-dose data for subsequent modeling activities. In the population PK analysis, we used the Michaelis–Menten (MM) approximation of the full TMDD model. The km value was estimated from phase 1 and 2 data where a broad dose range was studied (70, 105, and 140 mg every 2 weeks [Q2W] and 280, 350, and 420 mg once monthly [QM]), enabling a robust estimate of km. Phase 3 studies evaluated optimal doses selected from phase 2 (140 mg Q2W and 420 mg QM). The inclusion of 2 doses required fixing km because the extent of nonlinearity is less evident at these high doses. The MM approximation of the full TMDD model is appropriate to describe the system when target concentrations are small relative to the free drug concentrations [25].

Stochastic Approximation Expectation Maximization (SAEM) and Monte Carlo Importance Sampling (IMP) methods [26] were used for structural model development. The SAEM method was conducted in 2 phases: i) burn-in phase until convergence criteria based on evaluation of objective function, thetas, sigmas, and all omega elements were achieved, followed by ii) accumulation phase. The IMP method was evaluated for the purpose of obtaining standard error estimates for each parameter and ensuring stability across independent fits to the model.

Because approximately 19% of the serum free evolocumab data were below the LLOQ, the M3 method [27–29] was used to analyze serum concentrations below LLOQ.

The between-subject variability (BSV) in the model parameters was assumed to follow a log-normal distribution. BSV was implemented as a full-block variance matrix for random effects on all parameters except for k_a_ and k_m_, as these 2 parameters were fixed from previous steps. The residual variability of the PK model was assumed to have both additive and proportional components.

In the first step, covariates were evaluated univariately. As discussed in the literature [30], statistical significance does not necessarily predict clinical importance; instead, inferences about clinical importance driven by estimated magnitude of effect and associated precision may be more appropriate. Using a similar approach [30], during univariate covariate analysis, point estimates for covariate effects were estimated for each covariate. Covariates were considered significant if the 95% confidence interval (CI) of the point estimate of covariate effect did not include 0 for continuous covariates or did not include 1 for categorical covariates. The 95% CI was calculated based on the standard error estimates following the IMP step. Given the stochastic nature of the SAEM and IMP methods, the change in objective function could not be used as statistical significance criteria for covariate inclusion and exclusion. If the covariate in the univariate analysis was found to be significant based on the above criterion, then the covariate was included in the model with its estimated effect fixed from that step. This step was continued for the rest of the covariates univariately on top of the existing significant covariates in the model, until a full model was obtained. A final model including all significant covariates in the model building process allowing all covariate estimates to be estimated at the final step was used to account for any interacting effects. This final model was used for all simulation and prediction.

Continuous covariates were modeled according to the general equation:

$${\text{P}}_{j} = TVP \cdot \left( {\frac{{{\text{cov}}_{j} }}{\text{cov}}} \right)^{\varTheta } \cdot \exp (\eta_{j} )$$

Categorical covariates were modeled according to the general equation:

$${\text{P}}_{j} = TVP \cdot {\varTheta }^{\text{cov}_{j}} \cdot \exp (\eta_{j} )$$

where P_*j*_ is the individual model parameter for the jth subject, TVP is the typical value of the model parameter P, cov_*j*_ is the individual’s value of the covariate, cov is the population median value of the covariate, Θ is the magnitude of the covariate effect, and η_*j*_ is an independent and normally distributed random variable with mean 0 and variance ω^2^.

Covariates of interest included demographic parameters (body weight, sex, age, and race), concomitant medications (statins and ezetimibe), laboratory variables (baseline PCSK9), and disease state (heterozygous familial hypercholesterolemia [HeFH] and renal function). Of the race groups, only the African American group contained enough individuals to estimate covariate effects. The statin covariate represents patients on a statin only and no other comedication because statin comedication was a particular covariate of interest. The ezetimibe covariate includes all patients on ezetimibe, regardless of comedications. The dataset did not include enough patients on ezetimibe alone (< 3%) for an accurate measurement of the independent effect. For the PK model, any duration of administration of comedication was considered a covariate. Though for monoclonal antibodies renal elimination may be unlikely, potential changes in PK due to varying extents of renal impairment is a critical piece of information for the label. Therefore, a population PK approach similar to that performed for other monoclonal antibodies [31–33] was undertaken to rule out any possibilities of renal effect. The effect of renal function on PK was evaluated using both Cockcroft-Gault creatinine clearance (CrCL) and the Modification of Diet in Renal Disease (MDRD) measures. Across 26 placebo-controlled and active-controlled clinical trials, 0.1% of patients treated with at least one dose of evolocumab tested positive for binding antibody development. None of these patients tested positive for neutralizing antibodies. There was no evidence that anti-drug binding antibodies affected the PK profile, clinical response, or safety of evolocumab. Therefore, the incidence of anti-evolocumab binding antibodies is low, and not deemed necessary to evaluate in this analysis [34]. In addition, various analyses showed that evolocumab produced similar lipid-lowering effects in patients with and without diabetes, and hence not deemed a clinically relevant covariate in this analysis [35–37]. Albumin range was expected to be narrow for this population, hence not formally evaluated as a covariate. All covariates evaluated were baseline only.

When evaluating categorical effects, in order to ensure an adequate number of patients per category, categorical covariates with 5% or greater prevalence in the population data set of phase 1 and 2 data were evaluated for covariate effects. Of the race groups, only the African American group contained more than 5% of the population dataset to attempt to estimate covariate effects against a reference White patient. The ezetimibe covariate included all patients taking ezetimibe, regardless of lipid-lowering concomitant medications. Of 148 patients taking ezetimibe in the phase 1 and 2 PK model, 117 (79%) were also taking a statin. Thus, the ezetimibe covariate most generally represented a combination therapy covariate (hereafter notated as statin + ezetimibe). Patients with missing body weight, CrCL, or MDRD values were imputed to the mean values, and patients with missing baseline PCSK9 concentrations were excluded from analyses that included baseline PCSK9 as a covariate. The effect of each demographic and renal function covariate was estimated against CL, V, and V_max_, and the concomitant medications, laboratory variables, and HeFH were estimated against V_max_ due to their possible relationships to unbound PCSK9 concentrations [8, 19, 38].

Finally, data from 5 phase 3 studies were used to update the popPK model. The observed phase 3 data were overlaid on the model predictions from the phase 1 and 2 model to ensure that no major differences were evident between the phase 3 PK data and the phase 1 and 2 PK data. Because of the sparseness of the phase 3 data and the use of only 2 dosing regimens in phase 3, estimates of V_max_ and k_m_ were fixed to the phase 1 and 2 model parameter estimates. Similarly, additional covariates were not tested due to the sparseness of the data and potential for shrinkage [39]. Of 404 patients taking ezetimibe in the final PK model, 377 (93%) were also taking a statin; thus, the ezetimibe covariate continued to most generally represent a statin + ezetimibe combination therapy covariate.

### Exposure–response analysis

The longitudinal PK/PD relationship in an indirect response model using results from Phase 1a and 1b studies has been reported elsewhere [12]. In phase 2 and phase 3 studies, the primary efficacy endpoint was the mean of weeks 10 and 12 and week 12 LDL-C reduction. The exposure–response analysis based on the primary endpoints was best described by an Emax model, rather than the longitudinal response, which would be best described by the indirect response model. Hence, a week 8–12 exposure–response model was used to characterize the relationship between evolocumab exposure and LDL-C at the mean of weeks 10 and 12 (LDL_mean,wk10&12_) for combined data from all the phase 2 studies. LDL_mean,wk10&12_ was a surrogate for the time-averaged effect (TAE) on LDL-C over weeks 8–12. It represented a time-averaged LDL-C reduction over the dosing interval and a comparable measure across dosing regimens Q2W or QM. Further, the absolute LDL-C values were measured clinically and are considered the raw data collected from the studies. We confirmed the appropriateness of the model by transforming the data to  % change from baseline and evaluating diagnostic plots of DV vs PRED, and WRES vs PRED. Evolocumab area under the concentration–time curve from week 8 to 12 (AUC_wk8–12_) was used as the exposure metric, because this represented exposure at steady state during the same time period that the response variable was assessed. AUC_wk8–12_ for each patient in the phase 2 studies was predicted from the individual parameter estimates from the phase 1 and 2 PK model. Using the predicted AUC_wk8–12_ eliminated the residual error in the PK, which is a key assumption in the predictor variable for exposure–response relationships. Placebo response was evaluated but was not included in the model, because it was found to be negligible and did not influence the model parameters. The modeled data included the observed predose (days –13 and 0) LDL-C measurements and LDL_mean,wk10&12_.

The model took the form:

$$Eff = \frac{{E_{\hbox{max} } \cdot AUC_{wk8 - 12} }}{{(EC_{50} \cdot REG^{i} ) + AUC_{wk8 - 12} }}$$

Both (1) additive and (2) proportional-effect models were tested to relate the effect size to the baseline of LDL-C:

1 $$Y = BASL + Eff$$

2 $$Y = BASL \cdot (1 + Eff)$$

where Y is the predicted response for any AUC_wk8–12_, BASL (mg/dL) is the baseline LDL-C concentration informed by the predose LDL-C measurements, E_max_ (mg/dL or a unit-less fraction) is the theoretical maximum evolocumab response for the mean of weeks 10 and 12, EC_50_ is the AUC_wk8–12_ required to achieve half-maximal response with evolocumab dosed Q2W, and *Eff* is the effect magnitude. A regimen effect (REG) was modeled as a multiplier on EC_50_ to account for the dosing interval differences between the Q2W and QM regimens. An indicator variable, *i*, with a value of 0 or 1, was used to indicate Q2W or QM regimens, respectively. If the 95% CI of the REG multiplier included 1, the EC_50_ of the QM regimen did not differ significantly from the Q2W regimen. This way, we could assess if regimen would have any impact on efficacy. The different EC_50_ values are a result of the difference in the time courses of target saturation between the dosing regimens, given the lack of kinetics in the exposure–response E_max_ model. A random effect on the baseline was included in the model assuming a log-normal distribution. Additive, proportional, and combined additive and proportional error models were considered to describe the residual variability. Data were fit using first-order conditional estimation method with interaction (FOCEI) followed by an IMP step for the purpose of obtaining standard error estimates for each parameter. A placebo effect was tested on Y.

Covariates of interest included concomitant medications (statins and ezetimibe), disease (diabetes and HeFH), and laboratory variables (baseline PCSK9). For the exposure–response model, only stable concomitant medication (> 4 weeks of administration before study day 1) was considered a covariate to ensure an accurate estimation of the effects of baseline concomitant medication use. The statin covariate represented patients taking only a statin and no other concomitant medication. The ezetimibe covariate included all patients taking ezetimibe, regardless of concomitant medications. Of 160 patients taking ezetimibe in the exposure–response model, 158 (99%) were also taking a statin; thus, the ezetimibe covariate most generally represented a combination therapy covariate. Patients with missing baseline PCSK9 concentrations were excluded from analyses that included baseline PCSK9 as a covariate. Concomitant medications and HeFH were estimated against both the baseline LDL-C concentration and *Eff*, and diabetes and laboratory variables were modeled against *Eff*.

Finally, the observed phase 3 data were overlaid on the model predictions from the phase 1 and 2 model to ensure that no major differences were evident between the phase 3 response data and the phase 1 and 2 response data. Because of the sparseness of the phase 3 data and the use of only 2 dosing regimens in phase 3, possibly leading to increased shrinkage in the updated PK model, the exposure–response model was not updated with data from phase 3. Similarly, additional covariates were not tested due to the sparseness of the data and potential for shrinkage. No liabilities in the results are anticipated as the patient population was similar between phase 2 and phase 3 studies; hence, it is not expected that inclusion of additional patients from phase 3 would lead to identification of any additional covariates.

For comparisons of outcomes across significant covariates, an 84 kg male patient (mean weight of the patients in this analysis) with hypercholesterolemia, not taking other lipid-lowering medications and with baseline PCSK9 of 5.9 nM (425 ng/mL), was considered as the reference patient.

### Model-based simulation

Forest plots were generated based on the simulated parameter, incorporating the uncertainty for each significant covariate condition on the final covariate models. Parameter sets for 1000 individuals were constructed based on the variance–covariance structure for the thetas of the final covariate models using the simpar function built into the NONMEM HPC [40]. These simulations did not include BSV or residual variability. Outcomes of interest for each individual, AUC_wk8–12_ and LDL_mean,wk10&12_, were simulated under the significant covariate conditions of the final covariate models. Thus, the outcome for each individual under a covariate condition could be compared to the “same” individual in the reference (e.g., without the covariate condition). In this way, the geometric mean of the change in outcome relative to baseline for each covariate condition could be calculated across 1000 individuals. These were plotted along with the 95% CI of the geometric mean [41].

## Results

The final dataset was pooled from 5474 patients across phase 1, 2, and 3 studies to represent the intended target population. Of these patients, 3414 receiving evolocumab were included in the popPK analysis, and 1314 patients across 4 phase 2 studies were included in the exposure–response analysis. The popPK analysis dataset contained 16,179 evolocumab concentrations. Table 2 shows patient baseline characteristics.

**Table 2:** Patient characteristics

Covariate	Category	Phase 1 and 2 (N = 1045)	Phase 1, 2, and 3 (N = 3414)
Age, years	Mean (standard deviation) [range]	56 (58) [18–80]	57 (58) [18–80]
Body weight, kg	Mean (standard deviation) [range]	84.2 (82) [42–173]	84.2 (83) [41–175]
Baseline proprotein convertase subtilisin/kexin type 9, ng/mL	Mean (standard deviation) [range]	433 (409) [150–1233]	402 (375) [15.5–1233]
Baseline albumin, g/dL	Mean (standard deviation) [range]	4.0 (0.3) [2.6–5.6]	4.3 (0.4) [2.6–5.6]
Sex, n (%)	Male	488 (47)	1708 (50)
	Female	557 (53)	1706 (50)
Statin therapy, n (%)	None	415 (40)	938 (28)
	Atorvastatin	160 (15)	1170 (34)
	Rosuvastatin	172 (17)	731 (21)
	Simvastatin	244 (23)	497 (15)
	Lovastatin	11 (1)	12 (0)
	Pravastatin	32 (3)	41 (1)
	Pitavastatin	1 (0)	3 (0)
	Fluvastatin	8 (1)	13 (0)
Concomitant medication, n (%)	Ezetimibe	143 (14)	404 (12)
	Omega-3 fatty acid	35 (3)	159 (5)
	Bile acid sequestrants	3 (0)	29 (1)
	Other lipid-lowering therapy	4 (0)	8 (0)
	Niacin	1 (0)	8 (0)
	Fibrates	1 (0)	2 (0)
Disease state, n (%)	Diabetes	108 (10)	386 (11)
	Heterozygous familial hypercholesterolemia	108 (10)	318 (9)
Race/ethnicity, n (%)	White or Caucasian	885 (85)	2984 (87)
	Black or African American	96 (9)	222 (7)
	Hispanic or Latino	11 (1)	11 (0)
	Asian	34 (3)	129 (4)
	American Indian or Alaska native	5 (0)	10 (0)
	Native Hawaiian or other Pacific Islander	5 (0)	8 (0)
	Other	9 (1)	47 (1)
	Multiple	0 (0)	3 (0)

A 1-compartment model with linear and nonlinear elimination pathways characterized the PK of unbound evolocumab after IV and SC dosing (Fig. 1). In the covariate analysis based on data from phase 1 and 2 studies, body weight, female sex, concomitant use of a statin, concomitant use of statin + ezetimibe, and baseline PCSK9 concentration emerged as statistically significant covariates on unbound evolocumab PK. With the inclusion of covariates in the popPK model, BSV on CL and V_max_ was reduced (from 87.4% to 75.5% and from 73.9% to 50.4%, respectively), but BSV on V was not reduced (from 28.3% to 28.1%). Statin, statin + ezetimibe, and HeFH were statistically significant covariates on the exposure–response relationship. The statin effect accounted for differences between patients taking or not taking statins. Any influence of high- vs low-intensity statins and their impact on PCSK9 was addressed by including baseline PCSK9 levels in the model.

**Fig. 2:**
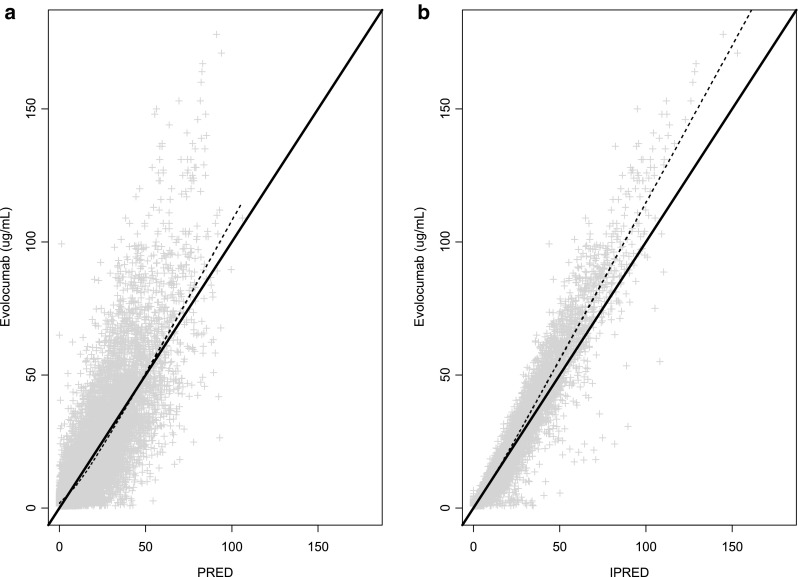
Observed and model-predicted serum evolocumab concentrations. Solid line: line of unity; dashed line: locally weighted scatterplot smoothing (LOWESS); *PRED* population predicted concentration; *IPRED* individual predicted concentration

**Fig. 3:**
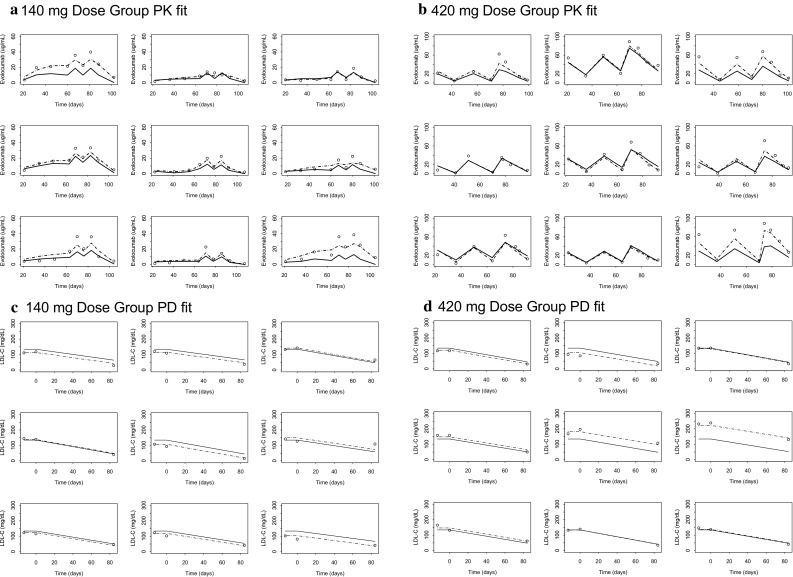
PK and PD individual model fits for representative patients. Points: observations; solid line: population prediction (PRED); dashed line: individual prediction (IPRED)

**Fig. 4:**
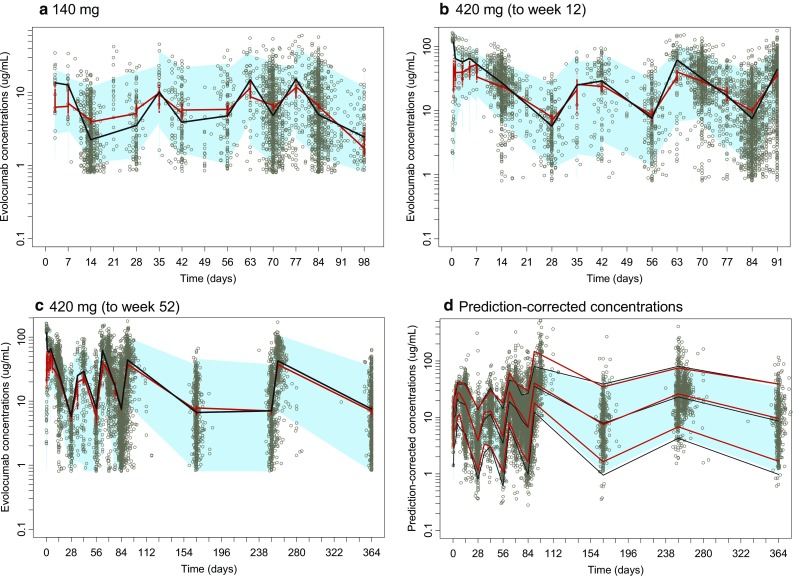
Time-course of model-predicted and observed serum evolocumab concentrations after doses of 140 mg SC Q2W (6 doses) and 420 mg SC QM (3 doses). Simulations were performed (number of trials = 100) on the entire dataset. Dots: observed evolocumab serum concentrations. Panels a, b, and c: blue shaded area: 90% prediction interval of simulated evolocumab serum concentration–time profile, and red line is predicted median, whereas black line is observed median. Panel d: The solid red lines represent the 95th (upper red line), 50th (middle red line), and 5th (lower red line) percentiles of the observed prediction-corrected serum concentration. The observed prediction-corrected plasma concentrations are represented by grey circles. The black lines (upper: 95th, middle: 50th, and lower: 5th) represent the simulation-based prediction, and the surrounding semitransparent blue field represents a simulation-based 90% prediction interval for the corresponding simulation-based prediction intervals. *Q2W* once every 2 weeks; *QM* once monthly; *SC* subcutaneous

**Fig. 5:**
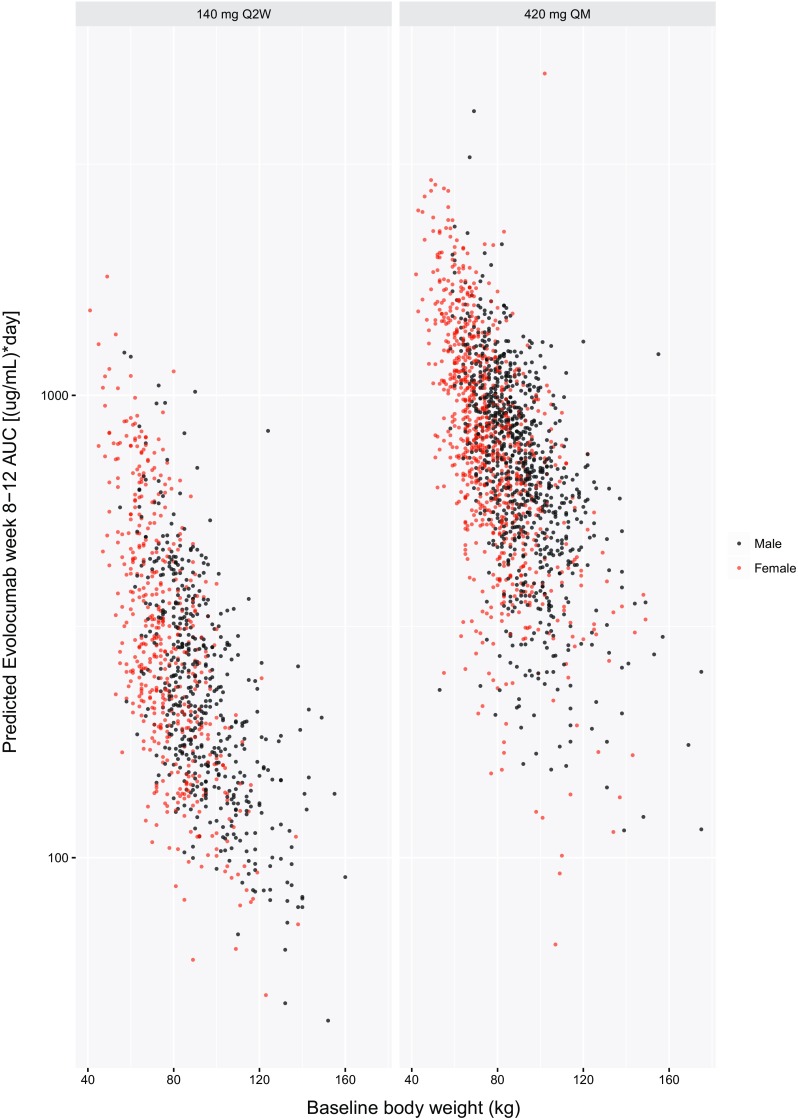
AUC week 8–12 versus body weight for all patients by sex

The updated phase 3 popPK model, including all the significant covariates from the phase 1 and 2 model, fit the data adequately, and parameter estimates were similar to those from the phase 1 and 2 model (Table 3). Both structural model parameters and residual variability parameters were estimated with good precision, as measured by a relative standard error (RSE) of < 5%. Covariate effects also were precisely estimated (RSE < 8%), with the exception of the body weight effects on CL and V_max_, which were 30.4% and 33.0%, respectively. The precision of the body weight covariate effects on CL and V_max_ may have been influenced by the high correlation between individual random effects on CL and V_max_ (Table 4). Baseline PCSK9 concentrations (~ 400 ng/mL) were small relative to mean observed unbound evolocumab C_max_ of 18.6 or 59 µg/mL after 140 mg SC Q2W or 420 mg SC QM, respectively [34]. In the MM TMDD model approximation, k_m_ represented the ratio of the sum of the complex internalization rate and dissociation rate constant to the drug-target dissociation rate constant. Because k_m_ and K_d_ values reflect different processes, it would not be appropriate to fix k_m_ to the measured in vitro K_d_. The large differences in k_m_ and in vitro K_d_ most likely reflect impact of rapid elimination of the drug-target complex on the k_m_ value. Precision of the diagonal elements of the BSV estimates was good (RSE < 10%). Goodness-of-fit plots (Fig. 2) and individual model fits (Fig. 3) showed the adequacy of the model to describe the data and the absence of systematic bias. The goodness-of-fit plot for the population PK model suggest that in general, there was a good agreement between the observed and model-predicted evolocumab serum concentrations, although some high concentrations were under-predicted. This is particularly evident in the QM dosing regimens. During the model building process, an attempt was made to use the quasi steady state approximation of the TMDD model. However, due to limited data in the lower dose ranges, the model was over-parametrized, and hence a simpler approach using the MM approximation of TMDD model was adopted. This under-prediction was not considered important as the outcomes of interest for the PK model, AUC_wk8–12_ and the week 12 trough concentration, were well predicted. With the exception of a negligible number of outliers who missed a dose, the week 8-12 AUCs for the available observed data in phase 2 substudy patients are accurately predicted by the model. As noted above, it is possible that use of a 1 cmt model instead of a 2 cmt model produces some bias in the model. However, due to data being mostly subcutaneously administered, this could not be ascertained during model building. Results of the visual predictive checks (Fig. 4) confirmed the ability of the model to describe the time-course of evolocumab concentrations and its associated variability in the target population. For patients in the phase 2 PK substudies (in which a PK time-course was collected), observed AUC_wk8–12_ was well-predicted by the model, providing another confirmation of the model adequacy. AUC_wk8-12_ was found to be correlated with body weight and sex (Fig. 5).

**Table 3:** Parameter estimates for population pharmacokinetics model

Parameter (definition)	Units	Estimate (% relative standard error)	Between subject variability (% relative standard error)	Shrinkage
F (subcutaneous bioavailability)	%	0.72 (FIXED)	0%	–
k_a_ (absorption rate constant)	day^−1^	0.319 (FIXED)	74.6% (FIXED)	48.4%
CL (linear clearance)	L/day	0.105 (2.18%)	54.3% (3.20%)	47.6%
Body weight exponent		0.276 (30.4%)		
V (volume of distribution)	L	5.18 (1.15%)	28.3% (3.27%)	25.2%
Body weight exponent		1.04 (4.05%)		
Female exponent		1.11 (1.42%)		
V_max_ (nonlinear clearance capacity)	nM/day	9.85 (FIXED)	31.1% (3.54%)	43.8%
Body weight exponent		0.145 (33.0%)		
Statin exponent		1.13 (1.02%)		
Statin + ezetimibe exponent		1.20 (1.59%)		
PCSK9 baseline exponent		0.194 (7.47%)		
k_m_ (concentration of half-maximal nonlinear clearance)	nM	27.3 (FIXED)	0% (FIXED)	–
Residual proportional error	%	0.282 (1.12%)	–	
Residual additive error	nM	5.41 (2.50%)	–

**Table 4:** Parameter estimates for exposure–response model

Parameter	Units	Estimate (% relative standard error)	Between subject variability (% relative standard error)	Shrinkage
Baseline LDL-C	mg/dL	150 (0.92%)	20.0% (2.26%)	7.87%
Statin exponent		0.797		
Ezetimibe exponent		0.768		
Heterozygous familial hypercholesterolemia exponent		1.28		
E_max_ (maximal change in LDL-C following evolocumab administration)	mg/dL	–99.7 (2.17%)	–	
Statin exponent		0.937		
EC_50_ (evolocumab exposure resulting in 50% of maximal effect	(μg/mL)·day	51.5 (9.79%)	–	
REG (regimen effect; once monthly relative to once every 2 weeks)	–	2.30 (10.3%)	–	
Residual proportional error	%	0 (FIXED)	–	
Residual additive error	mg/dL	19.3 (1.35%)

**Fig. 6:**
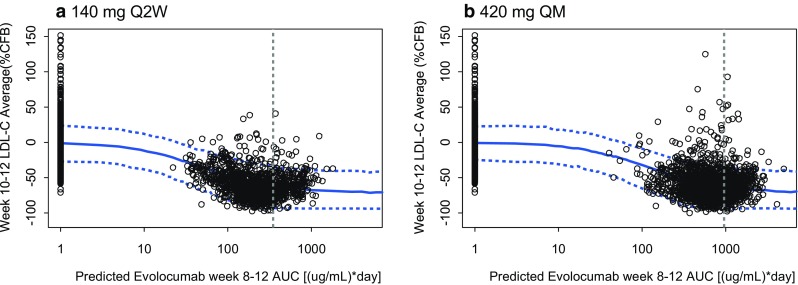
Observed data and 90% prediction interval for week 10 and 12 mean calculated LDL-C for phase 2 studies by weeks 8–12 evolocumab-predicted AUC. Prediction of the mean week 10 and 12 calculated LDL-C concentration, in percentage change from baseline, 50th (solid line) and 5th and 95th (dashed lines) percentiles. Simulations were performed for n = 2000 patients. Points: observed individual mean of weeks 10 and 12 LDL-C measurements. Vertical line: mean observed AUC_wk8–12_ in phase 2. *%CFB* percentage change from baseline; *AMG 145* evolocumab; *AUC* area under the concentration–time curve; *LDL*-*C* low-density lipoprotein-cholesterol; *Q2W* once every 2 weeks; *QM* once monthly

The exposure–response model characterized observed LDL-C_mean,wk10&12_ based on the individual predicted AUC_wk8–12_ from the PK model. The additive-effect exposure–response model fit the data and was selected. In the final exposure–response covariate model, concomitant use of a statin, concomitant use of statin + ezetimibe, and HeFH emerged as the biggest drivers of change in PCSK9 and evolocumab response (Table 4). Individual model fits of representative patients are shown in Fig. 3. Doses of 140 mg SC Q2W and 420 mg SC QM achieved approximately 80% of the model-predicted maximal reduction LDL_mean,wk10&12_. The model-predicted maximal reduction in LDL_mean,wk10&12_ was 99.7 mg/dL from a baseline value of 134 mg/dL, or a 66% reduction from baseline (Fig. 6). These doses of evolocumab led to a response near the plateau of the exposure–response relationship.

**Fig. 7:**
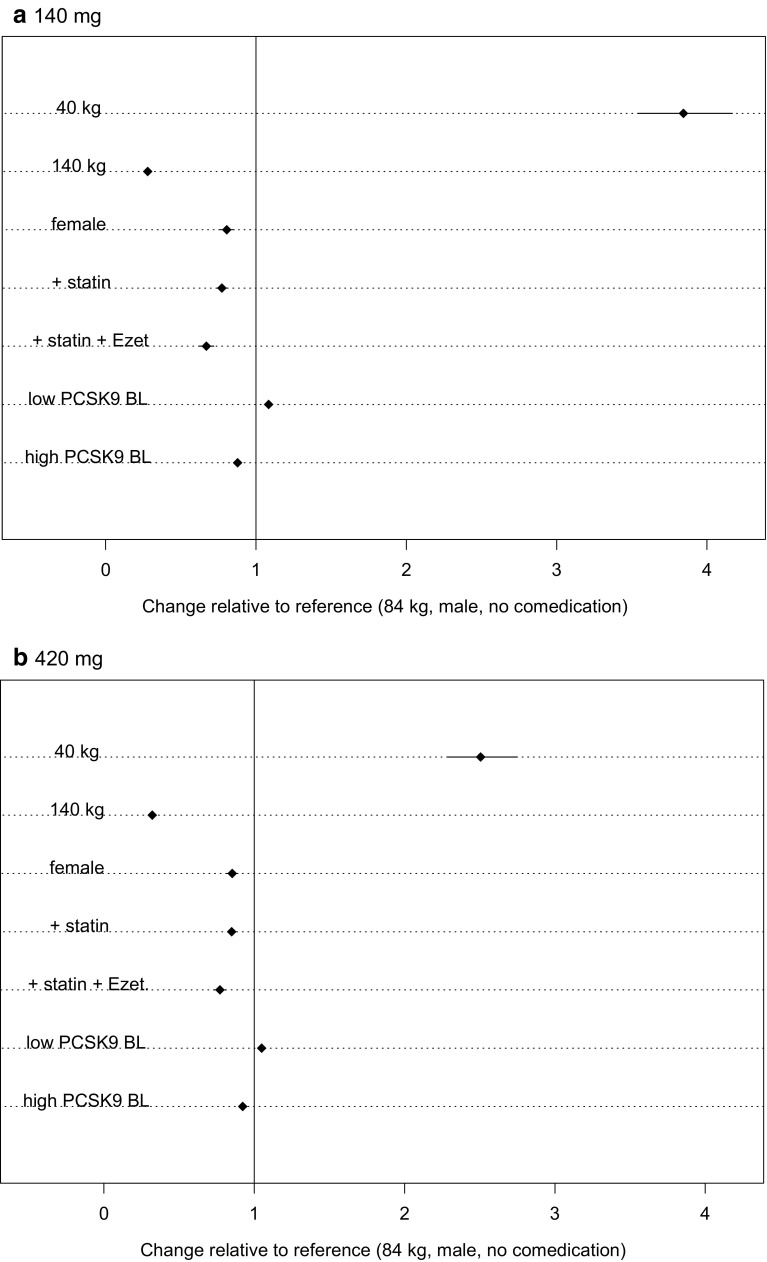
Forest plots of covariate effects with 95% CI for evolocumab AUC_wk8–12_ for 140 mg SC Q2W and 420 mg SC QM. The statin covariate represents patients only taking a statin and no other concomitant medication. The statin + Ezet covariate includes all patients on Ezet, regardless of concomitant medications. For patients in the pharmacokinetics model, 93% of those taking Ezet were also taking a statin; thus, the Ezet covariate most generally represents a combination (statin + Ezet) therapy covariate. *AUC* area under the time-concentration curve; *CI* confidence interval; *Ezet* ezetimibe; *PCSK9 BL* proprotein convertase subtilisin/kexin type 9 baseline (low, 4.8 nM [355 ng/mL]; high, 8.1 nM [599 ng/mL]); *Q2W* once every 2 weeks; *QM* once monthly; *SC* subcutaneous

**Fig. 8:**
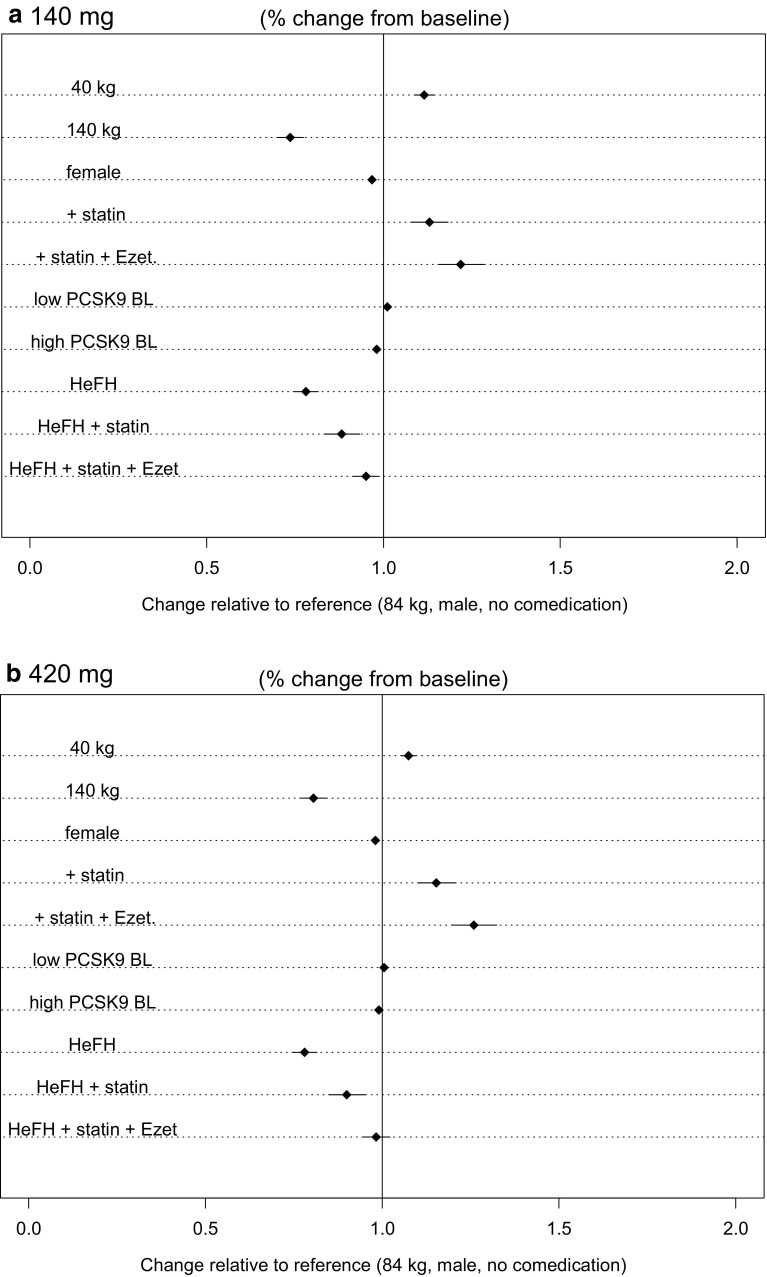
Forest plots of covariate effects with 95% CI for evolocumab week 10 and 12 mean calculated LDL-C lowering for 140 mg SC Q2W and 420 mg SC QM. *CI* confidence interval; *Ezet* ezetimibe; *HeFH* heterozygous familial hypercholesterolemia; *LDL*-*C* low-density lipoprotein-cholesterol; *PCSK9 BL* proprotein convertase subtilisin/kexin type 9 baseline (low, 4.8 nM [355 ng/mL]; high, 8.1 nM [599 ng/mL]); *Q2W* once every 2 weeks; *QM* once monthly; *SC* subcutaneous

As evident in the PK forest plots (Fig. 7), only body weight at the extremes (40 kg and 140 kg) seemed to markedly affect AUC_wk8–12_. Concomitant lipid-lowering medications such as statins and statins + ezetimibe showed a modest reduction in AUC_wk8–12_. In the response forest plots (Fig. 8), no covariates appeared to significantly modify evolocumab response. In these plots, a point estimate greater than 1.0 represented a stronger response (higher percentage change from baseline), and a point estimate less than 1.0 represented less of a response. Of note, all the HeFH patients in the dataset were taking a statin and/or ezetimibe, so simulations for a hypothetical HeFH patient not on lipid-lowering therapy were included for completeness but are not clinically relevant.

## Discussion

This popPK model was used to evaluate evolocumab PK from data collected during clinical development in phase 1, 2, and 3 studies. The 1-compartment model with linear and nonlinear elimination pathways was suitable to describe the time-course of serum unbound evolocumab concentrations following IV or SC administration of a single dose or multiple doses once every week, Q2W, or QM ranging from 7 to 420 mg in healthy subjects or patients with hypercholesterolemia. There was no evidence of time-dependent PK for evolocumab. Although the PK of a monoclonal antibody typically is described by a 2-compartment model [42], a 1-compartment model can be appropriate when the concentration data are mostly available following SC administration, and a relatively slower absorption phase masks the initial distribution phase of concentration decline that is observed following IV administration [42]. A 1-compartment PK model has been reported for some monoclonal antibodies such as efalizumab [43], golimumab [44], and ustekinumab [45].

Major factors affecting bioavailability of monoclonal antibodies following SC administration may include relative rates of presystemic catabolism and systemic absorption [46, 47]. The absolute bioavailability of evolocumab SC dose was fixed to 72% based on analysis of phase 1a densely sampled single-dose data, which is consistent with the reported values (24–95%) for other mono-clonal antibodies such as canakinumab, ustekinumab, and omalizumab [46–48].

The mechanisms of distribution of evolocumab are expected to be similar to other monoclonal antibodies. Accordingly, evolocumab typical V (BSV%) was 5.18 L (28%) and was similar to the values reported for efa-lizumab, basiliximab, and omalizumab [49–51]. Because evolocumab V is similar to typical plasma volume, this is consistent with a lack of extensive extravascular distribution.

Similar to other monoclonal antibodies [42], evolocumab is eliminated through a nonspecific (linear) pathway via the reticuloendothelial system and a target-mediated (nonlinear) pathway via PCSK9, which was implemented in the model as a capacity-limited (i.e., saturable) elimination process. CL was estimated to be 0.105 L/day, which is within the range of the reported CLs (0.071–0.535 L/day) of other monoclonal antibodies [42]. The V_max_ of 9.85 nM/day is equivalent to 7.7 mg of evolocumab eliminated per day through the nonlinear elimination route. Model predictions suggested that approximately 77% of evolocumab is eliminated through this nonlinear pathway for a single 140 mg SC dose, and approximately 51% for a single 420 mg SC dose. When the dose of evolocumab leads to serum concentrations below 0.4 µg/mL (approximately 10-fold below the k_m_ value), the nonlinear (and presumably target-mediated) elimination pathway is unsaturated, and the kinetics behave linearly. We have previously observed a TMDD relationship when simultaneously fitting unbound evolocumab and PCSK9 with the QSS TMDD model [12]. Dose proportional increases in evolocumab exposures were observed at doses greater than or equal to 140 mg SC when PCSK9 suppression was maintained over the dosing interval. Similarly, when the dose of evolocumab leads to serum concentrations above 40 µg/mL (approximately 10-fold above the k_m_ value), the proportion of the saturable elimination pathway becomes negligible when compared to the linear pathway, and the elimination kinetics behave linearly in this range as well. When the nonlinear elimination pathway is saturated, model parameters suggest a half-life of approximately 34 days. When the dose of evolocumab (or time-course of the concentration–time profile) leads to serum concentrations between 0.4 and 40 µg/mL, both the linear and nonlinear components affect the elimination kinetics. When the dose of evolocumab (or time-course of the concentration–time profile) leads to serum concentrations below 0.4 µg/mL, the nonlinear component becomes predominant, with an elimination half-life of approximately 1.9 days based on the ratio of V_max_ and k_m_. The model-predicted effective half-life for the clinical doses of 140 mg SC Q2W and 420 mg SC QM is 11.4 and 16.8 days, respectively, due to a combination of linear and nonlinear elimination pathways.

The exposure–response model showed robust reduction in calculated LDL-C across the evolocumab dose range studied. AUC_wk8–12_ accurately predicted the LDL_mean,wk10&12_, a surrogate for time-averaged LDL-C over weeks 8–12. This TAE is a complete measure of the effect of evolocumab and is a comparable measure across dosing regimens when PK and PD steady-state has been achieved. The model-predicted maximal reduction in LDL_mean,wk10&12_ was 99.7 mg/dL, or a 66% change from baseline, with an EC_50_ of 51.5 (µg/mL)·day for the Q2W dosing regimens and 118.5 (µg/mL)·day for the QM dosing regimens. Model predictions suggested that doses of 140 mg SC Q2W and 420 mg SC QM achieve approximately 80% of the model-predicted maximal reduction in LDL_mean,wk10&12_. Collectively, these data suggest that both the 140 mg SC Q2W and 420 mg SC QM dose regimens provide exposures within the plateau region of the exposure–response relationship, supporting their use in the clinic.

In the final PK covariate model, body weight, female sex, statin co-administration, statin + ezetimibe co-administration, and PCSK9 baseline emerged as statistically significant covariates on evolocumab PK. Statin co-administration, statin + ezetimibe co-administration, and HeFH disease state were found to be statistically significant covariates on the exposure–response relationship. Evolocumab exposure decreased with increasing body weight. However, reduction in LDL-C at the mean of weeks 10 and 12 of evolocumab administration was predicted to be within ± 26% of the reference patient (an 84 kg, male subject with hypercholesterolemia, not taking other lipid-lowering medications). Additionally, observed median LDL-C reduction at week 12 was similar across body weight quartiles (< 7% difference from lowest to highest quartile of body weight). Hence, dose adjustment based on body weight was not necessary. Although treatment with various statins was associated with lower unbound evolocumab exposure due to higher PCSK9 concentrations, reductions in LDL-C were comparable regardless of statin dose or intensity as reflected by changes in PCSK9 levels. Thus, intrinsic and extrinsic covariates, including statin co-administration, are not expected to have a clinically significant effect on the response to evolocumab treatment. Uncertainty-based simulation allows a quantitative understanding of the effect of covariates on drug response, including evaluation of “best” and “worst” case scenarios [52]. Such methodology is of particular use for therapeutics that exhibit nonlinear PK and/or exposure–response relationships, such as evolocumab, where simple extrapolations of the effects of changes in PK and PD parameters are not possible.

## Conclusions

In conclusion, by incorporating multiple levels of PK and exposure–response modeling and simulation, these analyses assessed the influence of intrinsic and extrinsic covariates as sources of variability in the PK and exposure–response relationships of unbound evolocumab. Model predictions suggested that evolocumab doses of 140 mg SC Q2W and 420 mg SC QM achieve similar reductions in calculated LDL-C and exposures within the plateau region of the exposure–response relationship, supporting clinical use of either dose. In addition, simulations suggested that the clinical response to evolocumab is consistent across differing clinical conditions. The range of responses based on intrinsic and extrinsic factors were not predicted to be sufficiently different from the reference patient to warrant evolocumab dose adjustment.
